# Effect of different pyrolysis temperatures on physico-chemical characteristics and lead(ii) removal of biochar derived from chicken manure†

**DOI:** 10.1039/c9ra08199b

**Published:** 2020-01-22

**Authors:** Yan Cuixia, Xu Yingming, Wang Lin, Liang Xuefeng, Sun Yuebing, Jia Hongtao

**Affiliations:** Key Laboratory of Original Environmental Pollution Prevention and Control, Ministry of Agriculture and Rural Affairs/Tianjin Key Laboratory of Agro-Environment and Agro-Products, Agro-Environmental Protection Institute, Ministry of Agriculture and Rural Affairs Tianjin 300191 China sunyuebing@aepi.org.cn +86-22-23618060 +86-22-23618061; College of Grassland and Environment Sciences, Xinjiang Agricultural University, Xinjiang Key Laboratory of Soil and Plant Ecological Processes Urumqi 830052 China jiahongtao2015@126.com

## Abstract

Biochar derived from chicken manure, as an effective metal adsorbent, was prepared through a pyrolysis method at different pyrolytic temperatures (200, 400, 600, and 800 °C). The physicochemical characteristics of chicken manure biochar (CMB) and its lead (Pb^2+^) adsorption mechanisms were studied by batch adsorption experiments, DTA/TGA, XRD, SEM-EDS, FTIR and an analysis of the composition of their mineral ash. Results showed that the best-fit for the Pb^2+^ adsorption data was achieved using a Langmuir isotherm and a pseudo-second-order model. The maximum adsorption capacities of Pb^2+^ increased with increasing of pyrolytic temperatures of the CMB, being 180.21, 200.80, 239.59, and 242.57 mg g^−1^, respectively, for CMB-200, CMB-400, CMB-600 and CMB-200. Although Pb^2+^ adsorption on CMB revealed that adsorption was controlled by multiple mechanisms, (*e.g.* surface complexation, ion exchange, surface precipitation, electrostatic attraction, physical adsorption, and co-precipitation), the ion exchange and surface precipitation played a dominant role in Pb^2+^ sorption. Using CMB for the removal of Pb from water is proposed as an effective, environmentally protective, novel approach.

## Abstract 2

Biochar derived from chicken manure, as an effective metal adsorbent, was prepared through a pyrolysis method at different pyrolytic temperatures (200, 400, 600, and 800 °C).

## Graphical abstract

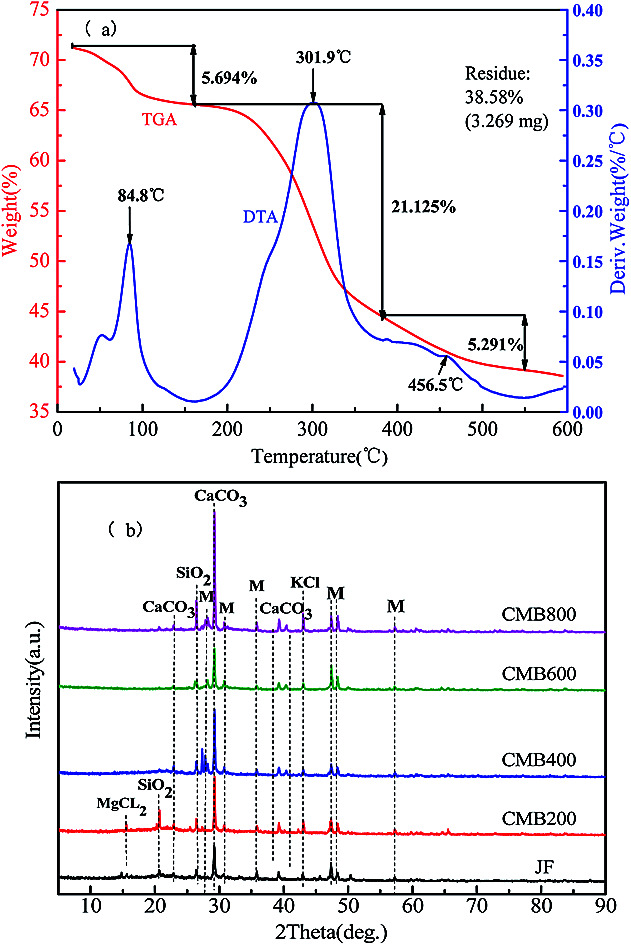

## 1 Introduction

Heavy metals are common pollutants in soil and water environments,^1^ and generally relate to human industrial and agricultural sources. Examples of such sources would be electroplating, mine tailings, industrial waste residues, sewage irrigation, leaded gasoline and paints, inadequate waste disposal, chemical fertilizers and pesticides, and spillage of petrochemicals.^2–6^ A nationwide survey carried out by the Ministry of Environmental Protection and Ministry of Land Resources showed that 19.4% of farmlands were heavily contaminated with toxic heavy metals. A study has found that people in the He Jian-tai region have elevated levels of Pb in their bodies, Pb was also detected in the reproductive and nervous systems of children.^7^ Studies have also shown that Pb toxicity in plants inhibits chlorophyll synthesis, decreases the germination rate, growth rate, and dry mass of roots,^8^ and causes seedling atrophy and slow growth.^9^ Therefore, the removal and/or remediation of Pb is imperative and emergent.

Biochar is a carbon-rich product produced by the pyrolysis of various waste materials including wood, poultry litter, and crop residues.^10–13^ Biochar possesses a developed pore structure, a relatively large surface charge, and a large specific surface area, is rich in surface functional groups, and is considered to be a good adsorption material.^14^ The application of biochar can improve soil fertility and may improve the physical, chemical, and biological characteristics.^15^ The adsorption potential of biochar for the removal of toxic metals from water is well reported. In recent years, biochar has attracted researchers with an interest in sorption materials because of its high sorption capacity for heavy metals. Biochar is relatively inexpensive and easy to obtain due to an abundance of raw materials (including agricultural crop and forestry residues, organic waste, and animal manure), and thus offers an optimal method for the removal of heavy metal contaminants.^16^ Numerous methods have been developed for the treatment of water polluted with Pb, including evaporation, dilution; chemical precipitation, electrolysis, ion exchange, reverse osmosis and membrane separation and other method. However, many of these processes have high operational costs and generate large volumes of toxic sludge and are therefore not suitable for large scale applications and so on. Compared with those, biochar adsorption seems to be more economical.^17^

With the continuous development of China's economy and the changes in people's diet, the number of intensive livestock and poultry farms and factories is increasing, yet there is a lack of fecal treatment facilities.^18^ Poultry and animal feces, and the original urine and fertilizers become waste, are currently non-point sources of rural pollution.^19^ The arbitrary stacking or the use of animal sourced waste can be an issue because of the potential environmental problems, such as, livestock and poultry wastewater, bad odors (NH, CH_4_S, H_2_S, and C_2_H_6_S *etc.*), propagation of pathogenic microorganisms, and the leaching of nitrates and other pollutants into groundwater.^20^ Lack of fecal treatment facilities ultimately results in ecological and environmental damage, and the wastage of precious agricultural resources. Thus, the preparation of biochar from chicken manure has been proposed as a way of reducing issues associated with livestock and poultry feces.^21^ The preparation of biochar from chicken manure for use in heavy metal pollution remediation not only solves the issue of livestock and poultry waste, but also prevents and eliminates waste pollution, thereby protecting ecology and the environment. To promote the sustainable usage of agricultural wastes like chicken manure and to control Pb^2+^ pollution, this study investigated CBC prepared from chicken manures at different pyrolysis temperatures. Characterization by scanning electron microscopy with transmission electron microscopy (SEM-TEM), Fourier-transform infrared (FTIR) spectroscopy, Raman spectroscopy (RS), and solid-state nuclear magnetic resonance (SSNMR) allowed the determination of the physicochemical properties of chicken manure biochar (CMB), as well as study of their Pb^2+^ removal capabilities from aqueous solutions, and of their Pb^2+^ adsorption mechanisms.

## 2 Materials and methods

### 2.1 Preparation of chicken manure biochar (CMB)

Biochar material was prepared from chicken manure through a slow pyrolysis process. Chicken manure samples were collected and then air-dried in the laboratory. Stones, feathers, and other impurities were removed. The characteristics of chicken manure were pH 6.5, OM 67.5%, total N 17.0 g kg^−1^, total P 6.3 g kg^−1^, and total K 7.6 g kg^−1^. The dried chicken manure samples were pulverized at high speed and sieved with a 200-mesh sieve before being dried in an oven at 75 °C for 12 h. The dried samples underwent slow pyrolysis at 200, 400, 600, and 800 °C for 2 h in a pyrolyzer, and were purged with N_2_ before pyrolysis. The obtained CMB was then dried in an oven at 75 °C overnight. The basic properties of the chicken manure biochar are summarized in Table 1.

**Table 1:** Indicators of pH, yield, ash, elemental compositions and atomic ratio by 200 °C of chicken manure biochar (CMB200), 400 °C of biochar (CMB400), 600 °C of biochar (CMB600) and 800 °C of biochar (CMB800)

Sample	pH	Yield (%)	Ash (%)	Elemental composition (%)				Atomic ratio (%)		
				C	H	O	N	O/C	H/C	(N + O)/C
CMB200	6.69	93.95	40.57	37.38	5.29	13.24	3.52	3.76	0.14	0.45
CMB400	7.29	70.96	44.78	22.01	2.62	28.34	2.25	12.60	0.12	1.39
CMB600	9.23	48.96	60.95	29.36	1.91	5.79	1.99	2.91	0.07	0.26
CMB800	10.11	44.82	64.63	30.35	1.34	1.67	2.01	0.83	0.04	0.12

### 2.2 Characterization of CMB samples

The pH value of the CMB samples was determined using a pH electrode to extract water (1 : 10 ratio). The elemental contents (C, H, O, N) of the CMB samples were determined using an Elemental Analyzer (Jena EA3000). The surface functional groups (before and after) of the samples were identified by Fourier transform infrared spectroscopy (FTIR Analyzer, Bruker Tensor), and the spectra of each sample was recorded in the 4000–500 cm^−1^ region. The mineral compositions of the CMB samples were analyzed using X-ray diffraction (XRD, Rigaku Ultima IV). The CMB samples were coated with gold and the structure morphologies and elemental compositions were observed using Scanning Electron Microscopy (SEM, SU3500) and Energy Dispersive Spectroscopy (EDS). The yield of each CMB sample was calculated by weighing the samples and using eqn (1).1
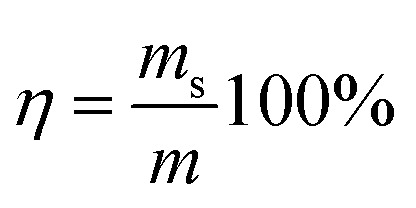
where *η* is the CMB yield, *m*_s_ (g) is the sample quality after pyrolysis, and *m* (g) is the sample mass before pyrolysis.

### 2.3 Pb^2+^ sorption experiments

#### 2.3.1 Adsorption kinetics

The sorption kinetics were determined by mixing 4.0 g of each CMB sample with 1000 mL of 0.01 mol L^−1^ NaNO_3_ solution containing 1000 mol L^−1^ Pb^2+^. The pH of the Pb^2+^ solution was adjusted to 5.0 with 0.1 mol L^−1^ HNO_3_ or NaOH solution prior to the sorption test and before being added to the CMB samples. The solutions were stirred on a magnetic mixer (200 rpm) at 25 °C for 24 hours. Samples were taken from each CMB at 0, 3, 5, 7, 15, 30, 60, 120, 240, 480, 720, and 1440 min, and the pH of each main solution was also determined. Each solution supernatant was filtered using a 0.45 μm membrane filter. The Pb^2+^ filtrates were analyzed by a flame atomic absorption spectroscopy (ASS) analyzer (2EEnit7009). The sorption kinetics were determined using eqn (2) for each CMB sample, and were then analyzed using pseudo-first-order and pseudo-second-order kinetics, as denoted by eqn (3) and (4), respectively.^22,23^2
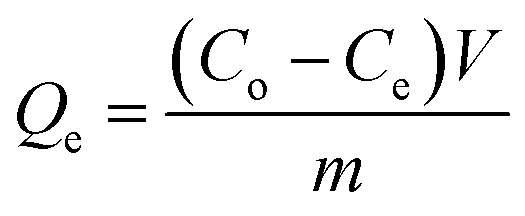

3 *Q*_t_ = *Q*_e_(1 − e^−*kt*^)

4
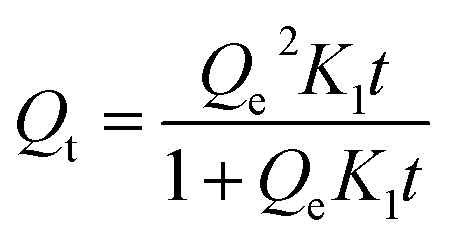
where *C*_0_ (mg L^−1^) and *C*_e_ (mg L^−1^) are the initial concentrations and equilibrium concentrations, respectively. *V* (L) is the volume of the solution, *m* (g) is the weight of CMB, and *Q*_t_ (mg g^−1^) and *Q*_e_ (mg g^−1^) represent the Pb^2+^ adsorption capacities at time *t* and equilibrium, respectively. *t* (h) represents the adsorption time, *K* (h^−1^) and *K*_1_ (mg g^−1^ h^−1^) are the pseudo-first-order and pseudo-second-order sorption rate constants, respectively.

#### 2.3.2 Adsorption isotherms

The isotherm experiments were conducted by placing 0.1 g of each CMB sample in 50 mL polypropylene tubes, and then by adding 25 mL of nine 0.01 mol L^−1^ NaNO_3_ solutions containing 10, 25, 50, 100, 200, 400, 600, 800, and 1000 mg L^−1^ Pb^2+^. The pH of each Pb^2+^ solution was adjusted to 5.0 with 0.1 mol L^−1^ HNO_3_ or NaOH solution before being added to each CMB sample. Polypropylene tubes were shaken using a temperature shaker incubator at 15, 25, and 35 °C for 12 h. After shaking, each solution supernatant was filtered using a 0.45 μm membrane filter. The Pb^2+^ filtrates were analyzed by AAS. Adsorption data of CMB samples were modelled using the Langmuir and Freundlich isotherms as expressed by eqn (5) and (6), respectively.^24^5
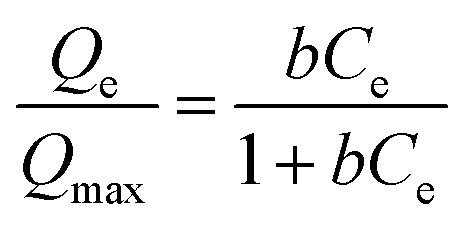

6 *Q*_e_ = *K*_f_*C*_e_^*n*^

where *Q*_e_ (mg g^−1^) is the adsorption capacity at equilibrium, *Q*_max_ (mg g^−1^) is the maximum adsorption capacity, *b* (L mg^−1^) is the interaction energy, *C*_e_ (mg L^−1^) is the equilibrium concentration, *K*_f_ (mg g^−1^ (mg L^−1^) *n*) is the coefficient of affinity, and *n* is the linearity constant.

#### 2.3.3 Effect of initial pH on Pb^2+^ adsorption

The effect of initial pH on the adsorption of Pb^2+^ was assessed by adding 0.1 g of each CMB sample to 25 mL of 400 mg L^−1^ Pb^2+^ at different pH values (2.5, 3.5, 4.5, 5.5, 6.5, 7.5, and 8.5) in 50 mL polypropylene tubes. The pH of each Pb^2+^ solution was adjusted using a 0.1 mol L^−1^ HNO_3_ or NaOH solution before being added to the CMB samples. The mixture was equilibrated on a temperature shaker incubator (200 rpm) at 25 °C for 12 h. Each solution supernatant was filtered using a 0.45 μm membrane filter. The Pb^2+^ filtrates were analyzed by ASS.

## 3 Results and discussion

### 3.1 Structural characterization of CMB

**Fig. 1:**
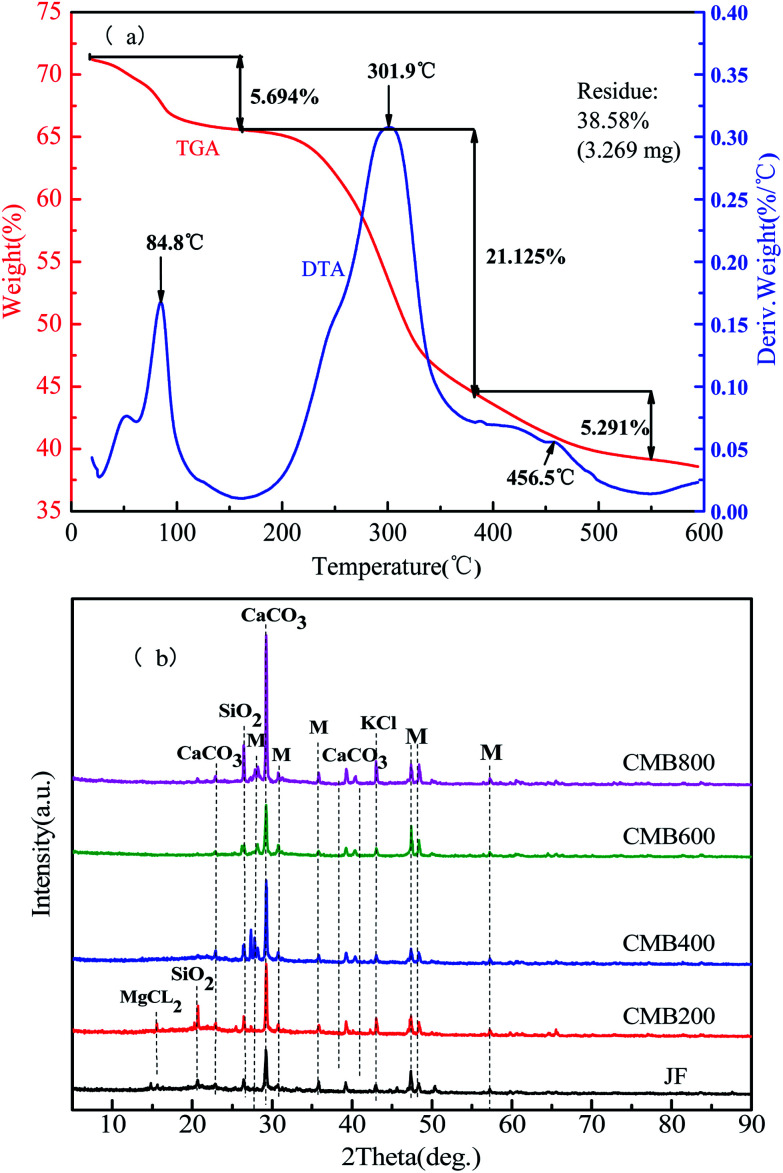
DTA/TGA (a) and XRD spectra analysis (b) of chicken manure biochar (CMB). Note: the symbols of M representation other substances.

Thermogravimetric Analysis (TGA) and Differential Thermal Analysis (DTA) curves can be obtained by the quality change rule, which uses the reaction conditions and the endothermic or exothermic properties of the sample after the temperature change. Fig. 1 shows the TGA and DTA curves obtained for chicken manure feedstock samples. In a nitrogen atmosphere, a heating rate of 10 °C min^−1^ was used until 600 °C was reached and maintained for 1 h in order to simulate the CMB preparation process. The DTA curve represents the endothermic and exothermic processes in the reaction. Fig. 1a shows the weight loss of the samples to 38.58% of the available inorganic residue.

The process of combustion weight loss of biomass can be divided into three stages. The first stage is between room temperature (25 °C) to 200 °C, when a weight loss of 5.694% represents loss of water evaporation and is shown by the peak positioned at 84.8 °C on the DTA plot. The second stage is between 200–350 °C, when a weight loss of 21.125% represents loss of hemicellulose, cellulose, part of the lignin, surface functional groups, and volatile combustion, and is shown by the peak positioned at 301.9 °C on the DTA plot. In the range of 350–600 °C, a weight loss of 5.291% is due to combustion of the residual organic components, at which time the carbon skeleton begins to disappear, and is shown by the peak positioned at 456.5 °C on the DTA plot.^25,26^

XRD is a widely used technique for analyzing the crystallinity of biomass and the structure of biochar.^27^ The XRD patterns of the CMB samples obtained at different pyrolytic temperatures in the 2*θ* values of 10–90° are shown in Fig. 1b. In the XRD spectra of the CMB samples shows that the main constituents are CaCO_3_ and SiO_2_, and that KCl and other substances are trace constituents. The lowest absorption intensity peaks are JF and CMB600. By observing the CMB200, CMB400, and CMB800 absorption peaks, it can be seen that there is an obvious increase in the intensity of CaCO_3_ with increasing carbonization temperature.^28^ Some components are vaporized and enriched on the surface of the biomass during pyrolysis, which results in a quantity of salt ions (K, Ca, and Si) being present in the four high temperature pyrolytic biochars, which are much higher than that in the JF sample. This can provide cations to promote ion exchange, and lead to a strong adsorption ability for heavy metals.^29^

### 3.2 Pb^2+^ sorption experiments

#### 3.2.1 Adsorption kinetics analysis

**Fig. 2:**
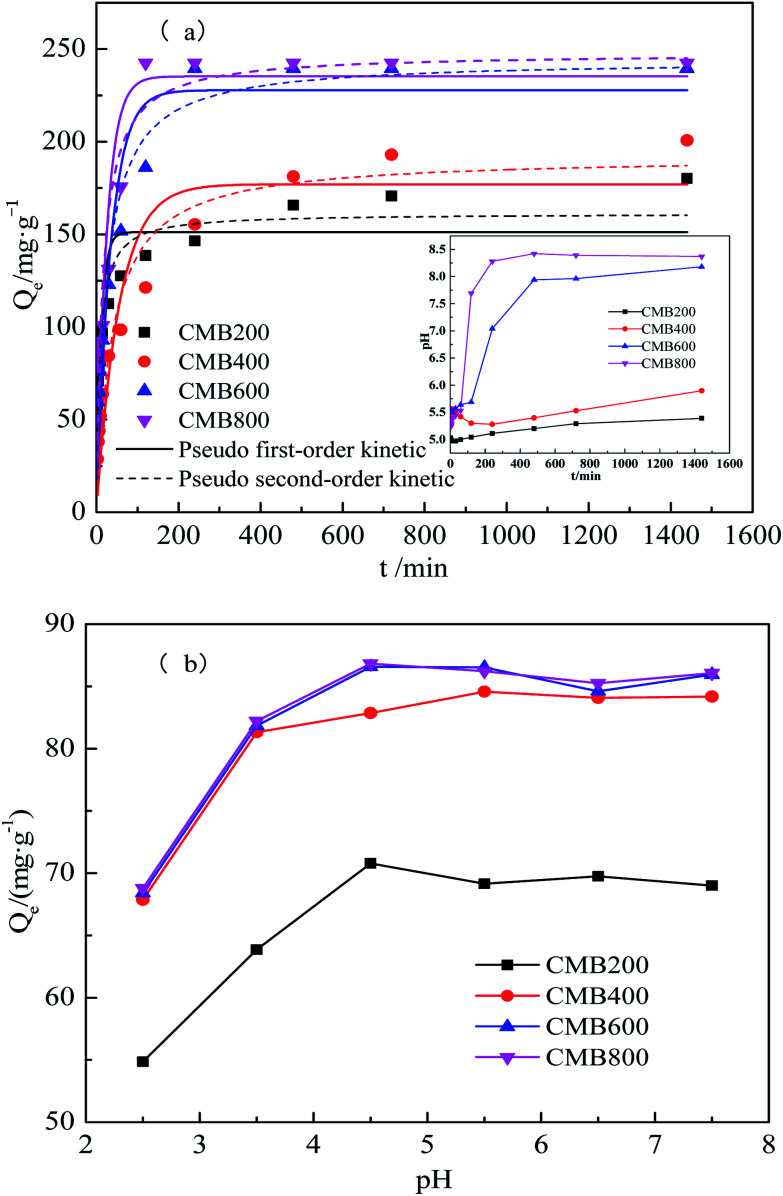
Kinetic adsorption curves (a) and the removal efficiency (b) of Pb^2+^ onto CMB.

The experimental data along with the pseudo-first-order model, the pseudo-second-order model, and the adsorption solution pH for the CMB samples are shown in Fig. 2a. The data show a rapid initial uptake for samples CMB800 and CMB600; the Pb^2+^ sorption required to reach apparent equilibrium was achieved in ∼120 min (pH 7.69) and ∼240 min (pH 7.04), respectively. Slower uptake meant that sorption equilibria was not reached until 480 min for CMB200 and CMB400 (pH 5.2 and 5.4, respectively). Rapid sorption equilibria suggests that electrostatic adsorption may contribute to Pb^2+^ sorption by the organic fraction of the biochar.^30,31^ The Pb^2+^ adsorption capacities were 180.21 mg g^−1^ (CMB200), 200.80 mg g^−1^ (CMB400), 239.59 mg g^−1^ (CMB600), and 242.57 mg g^−1^ (CMB800). These results can be attributed to CMB800 having more adsorption sites, and thus a better adsorption effect.^32^ The Pb^2+^ removal rates were 77.778%, 82.061%, 99.995%, and 99.997% for CMB200, CMB400, CMB600, and CMB800, respectively. As a result of the increasing carbonization temperature of the CMB samples, the Pb^2+^ removal rate and adsorption capacity were enhanced. Increased carbonization temperature also enhanced the surface area and porosity, thus resulting in faster rate of adsorption.^33^

Table 2 shows the results of the pseudo first-order, pseudo-second order, Langmuir, and Freundlich models. Among the two kinetic models tested, the pseudo first-order/pseudo-second order models yielded coefficients of determination (*R*^2^) of 0.772/0.908, 0.868/0.944, 0.906/0.965, and 0.834/0.905, respectively. These results showed that pseudo-second order simulation the kinetics data more closely match the actual adsorption data. Table 2 presents the pseudo-second order simulation the kinetics data that are close to the actual adsorption data, which provides a better fit for the entire range of the kinetic calculations. This also shows that the chemical adsorption process dominated in the adsorption of Pb^2+^,^34^ as reported previously.^35^

**Table 2:** Parameters of pseudo first-order kinetics, pseudo second-order kinetics models, Langmuir models and Freundlich models for Pb^2+^ sorption of CMBs

*T*/°C		Sample											
		CMB200			CMB400			CMB600			CMB800		
		Parameter 1	Parameter 2	Parameter 3	Parameter 1	Parameter 2	Parameter 3	Parameter 1	Parameter 2	Parameter 3	Parameter 1	Parameter 2	Parameter 3
	Actual equilibrium	*Q*_e_ = 180.2120	—	—	*Q*_e_ = 200.8040	—	—	*Q*_e_ = 239.5871	—	—	*Q*_e_ = 242.5733	—	—
	First-order	*Q*_e_ = 151.2012	*K*_1_ = 0.0870	*R* ^2^ = 0.7717	*Q*_e_ = 177.0918	*K*_1_ = 0.0180	*R* ^2^ = 0.8683	*Q*_e_ = 227.8398	*K*_1_ = 0.0287	*R* ^2^ = 0.9057	*Q*_e_ = 235.2942	*K*_1_ = 0.0409	*R* ^2^ = 0.8343
	Second-order	*Q*_e_ = 161.2479	*K*_2_ = 7.2831	*R* ^2^ = 0.9077	*Q*_e_ = 192.0881	*K*_2_ = 1.3400	*R* ^2^ = 0.9438	Qe = 244.2430	*K*_2_ = 1.6671	*R* ^2^ = 0.9645	*Q*_e_ = 247.8756	*K*_2_ = 2.5389	*R* ^2^ = 0.9047
15	Langmuir model	*Q*_e_ = 164.5026	*K*_f_ = 1.7301	*R* ^2^ = 0.9818	*Q*_e_ = 140.6585	*K*_f_ = 3.6550	*R* ^2^ = 0.9801	*Q*_e_ = 234.4906	*K*_f_ = 2.1748	*R* ^2^ = 0.9957	*Q*_e_ = 321.0200	*K*_f_ = 1.5224	*R* ^2^ = 0.9972
	Freundlich model	*n* = 0.6078	—	*R* ^2^ = 0.9479	*n* = 0.5043	—	*R* ^2^ = 0.9173	*n* = 0.6227	—	*R* ^2^ = 0.9733	*n* = 0.6917	—	*R* ^2^ = 0.9891
25	Langmuir model	*Q*_e_ = 227.2943	*K*_f_ = 1.3783	*R* ^2^ = 0.9757	*Q*_e_ = 311.6891	*K*_f_ = 1.0232	*R* ^2^ = 0.9894	*Q*_e_ = 494.6137	*K*_f_ = 0.7086	*R* ^2^ = 0.9949	*Q*_e_ = 583.1292	*K*_f_ = 0.6129	*R* ^2^ = 0.9971
	Freundlich model	*n* = 0.6697	—	*R* ^2^ = 0.9471	*n* = 0.7313	—	*R* ^2^ = 0.9741	*n* = 0.8086	—	*R* ^2^ = 0.9873	*n* = 0.8337	—	*R* ^2^ = 0.9920
35	Langmuir model	*Q*_e_ = 403.3246	*K*_f_ = 1.0982	*R* ^2^ = 0.9843	*Q*_e_ = 526.1257	*K*_f_ = 1.1274	*R* ^2^ = 0.99	*Q*_e_ = 775.4458	*K*_f_ = 0.8603	*R* ^2^ = 0.9821	*Q*_e_ = 812.2154	*K*_f_ = 0.8234	*R* ^2^ = 0.9836
	Freundlich model	*n* = 0.7520	—	*R* ^2^ = 0.9681	*n* = 0.7729	—	*R* ^2^ = 0.9785	*n* = 0.8337	—	*R* ^2^ = 0.9735	*n* = 0.8411	—	*R* ^2^ = 0.9757

#### 3.2.2 Adsorption isotherms analysis

**Fig. 3:**
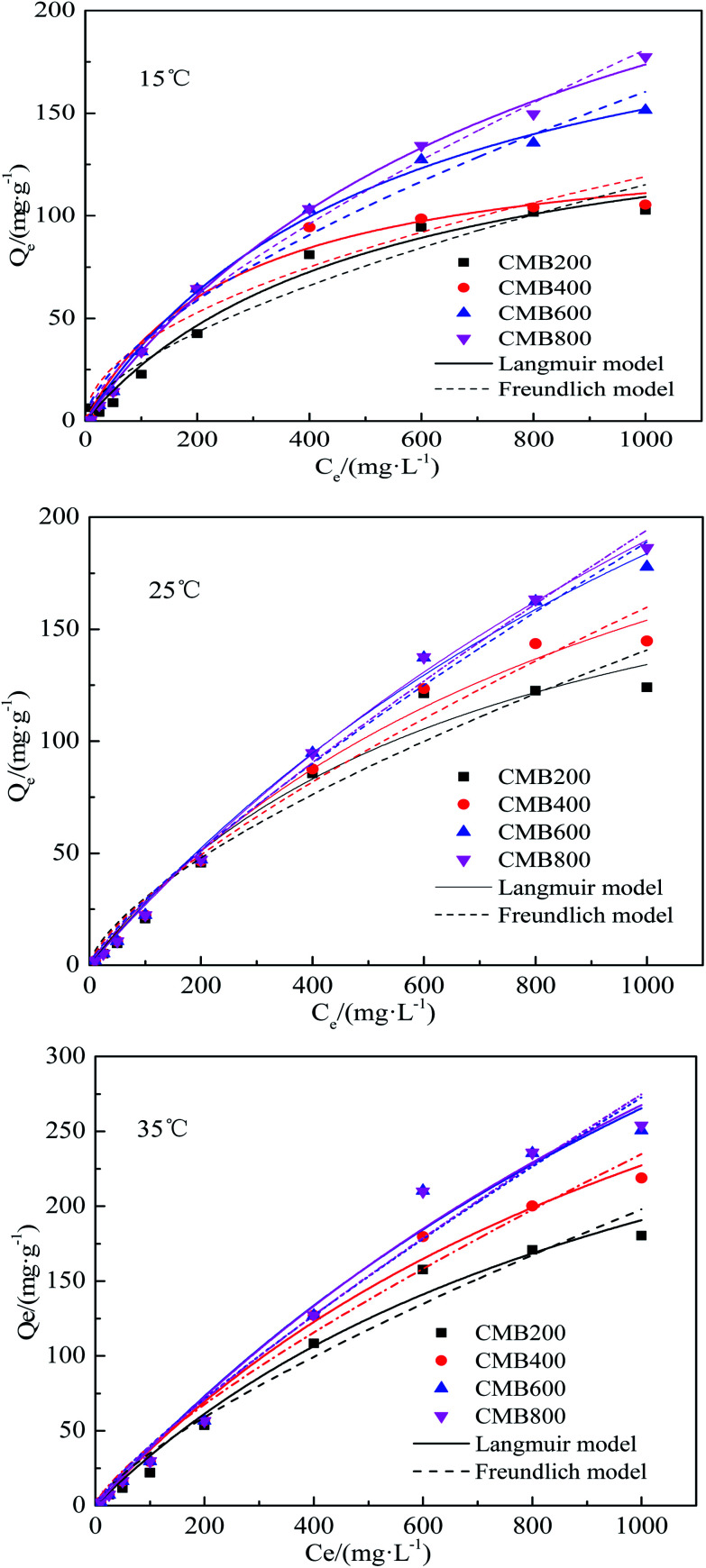
Adsorption isotherms curves of CMB for Pb^2+^.

The Langmuir and Freundlich models were used to model the sorption isotherms of the CMB samples at temperatures of 15, 25, and 35 °C (Fig. 3). The three isotherm experiment results indicate an increase in temperature greatly enhanced the Pb^2+^ sorption ability of the CMB samples. The equilibrium adsorption for Pb^2+^ increased with increasing initial concentration, which can be attributed to a lower initial solution concentration, whereby the biochar provides sufficient adsorption sites and active functional groups. The adsorption sites became gradually saturated and active groups were reduced with increasing initial Pb^2+^ concentration, thus the CMBs reached adsorption saturation.^36^

The Langmuir model simulated sorption capacities (*Q*_e_) (Table 2) for a temperature of 15 °C were 164.50, 140.66, 234.49, and 321.02 mg g^−1^ for CMB200, CMB400, CMB600, and CMB800, respectively. The sorption capacities (*Q*_e_) for a temperature of 25 °C were 227.29, 311.69, 494.61, and 583.13 mg g^−1^ for CMB200, CMB400, CMB600 and CMB800, respectively. Of the modeled temperatures, the adsorption effect was best at a temperature 35 °C, whereby the maximum sorption capacities were determined as 812.22 mg g^−1^ (CMB800) > 775.45 mg g^−1^ (CMB600) > 526.13 mg g^−1^ (CMB400) > 403.32 mg g^−1^ (CMB200). The sorption capacities at 35 °C were therefore 2–3 times those at 15 °C, and the CMB800 sorption capacity at 35 °C was 1–2 times of the other CMB samples at 35 °C. Thus, the result showed that the sorption capacity was enhanced with increasing pyrolytic temperature and adsorption temperature, and that the CMB800 has the best sorption capacity.

The adsorption equilibrium isotherm is important for describing how the adsorbate molecules are distributed between the liquid and the solid phases under an equilibrium state.^37^ Usually, the Langmuir model better describes adsorption on homogeneous surfaces, while the Freundlich model is better for heterogeneous surfaces.^38^Fig. 3 presents the Pb^2+^ sorption isotherms for the CMB samples. The fitting parameters are summarized in Table 2. The results show that the Langmuir model fitted the CMB200, CMB400, CMB600, and CMB800 (*R*^2^ = 0.982/0.976/0.984, 0.980/989/0.99, 0.995/0.995/0.982, and 0.997/0.997/0.984, respectively) sorption isotherm better than the Freundlich model (*R*^2^ = 0.947/0.947/0.968, 0.917/0.974/0.979, 0.973/0.987/0.974, and 0.989/0.992/0.976, respectively) for the adsorption temperature 15/25/35 °C. This also shows that for the adsorption of Pb^2+^, the chemical adsorption process dominated and suggests that adsorption was similar to that of monolayer adsorption.^39^

### 3.3 Effect of initial pH on Pb^2+^ adsorption

The effect of initial pH on the sorption of Pb^2+^ by the CMB samples for a pH range of 2.5–7.5 are shown in Fig. 2b. The pH value of metal ions in solution is an important parameter with regards to the adsorption behavior of transition metal ions on adsorbents. The values of pH affects not only the charge and surface structure of the adsorbents and the formation of transition metal ions, but also the interaction between adsorbents, the stability of metal complexes, the degree of ionization, and speciation in solution.^40,41^ The results show that the adsorption capacity increased with the increasing pH, as reported previously.^42^ This result can be attributed to the effect that increasing pH has on increasing the negative charges on the surface of the adsorbent, thereby increasing the adsorption of metals.^43^

Maximum adsorption capacities were 70.7914, 84.5789, 86.5618, and 86.8239 mg g^−1^ for CMB200, CMB400, CMB600, and CMB800 at an initial pH of 4.5, respectively. This shows that the CMB samples derived from the highest pyrolysis temperature had the highest adsorption capacity in comparison to that of the high pyrolysis temperature at all solutions for every pH. CMB samples had the best adsorption capacity for Pb^2+^ is in pH of 4.5–7.5 solutions.

### 3.4 The mechanism of Pb^2+^ removal by CMB

#### 3.4.1 SEM-EDS analysis

**Fig. 4:**
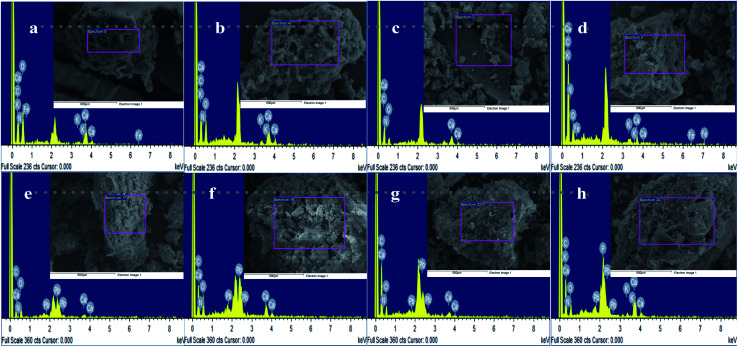
SEM-EDS comparative analysis of CMB before and after Pb^2+^ reaction: (a, b, c and d) SEM-EDS analysis of CMB before Pb^2+^ reaction; (e, f, g and h) SEM-EDS analysis of CMB after Pb^2+^ reaction.

Scanning electron microscope (SEM) images of CMB samples illustrate the morphological structural changes of the CMB samples following pyrolysis at different pyrolytic temperatures. Fig. 4 shows a SEM-Energy Dispersive Spectroscopy (EDS) comparative analysis of the CMB samples before and after treatment with Pb^2+^. The SEM-EDS analysis before CMB samples were treated with Pb^2+^ is shown in Fig. 4a, b, c and d. The rough surface and regular pores were observed on all biochars, whereas CMB samples had a developed pore structure. The pore diameter broadened with increasing pyrolytic temperature, which suggests that surface thermal decomposition gradually strengthened the CMB samples during pyrolysis, probably due to the organic matter decomposition of the biochar. The internal release of volatile gas and the formation of channel structures during pyrolysis have been reported to broaden the pore diameter,^27^ which could facilitate the adsorption of heavy metal ions.

Fig. 4e, f, g, and h show SEM-EDS images for the CMB samples after being treated with Pb^2+^. These show that the biochars maintained their original morphological structure and that they had rougher surfaces after adsorption in comparison to before adsorption, the surface and pores allow the adhesion of a large number of small particles, which can be Pb^2+^ or other impurities.

EDS images of before and after adsorption to CMB samples indicates the elemental analysis (Fig. 4 and Table S1†). Trends of a gradual increase in the C atomic ratio and a gradual decrease in the O atomic ratio with increasing pyrolysis temperature (200–800 °C), both before and after adsorption, were observed, however no such trends were observed for K and N atomic ratios. A gradual reduction in the Ca atomic ratio with increasing pyrolysis temperature before adsorption was observed, but after adsorption results reveal an irregular trend in the Ca atomic ratio and a gradual increase in the Pb^2+^ atomic ratio with increasing pyrolysis temperature. Each element atomic ratio was proportional to the difference between the CMB samples. The main constituent was Ca, followed by O, C, N, and trace amounts of K. The K content was zero for sample CMB600.

#### 3.4.2 FTIR analysis

**Fig. 5:**
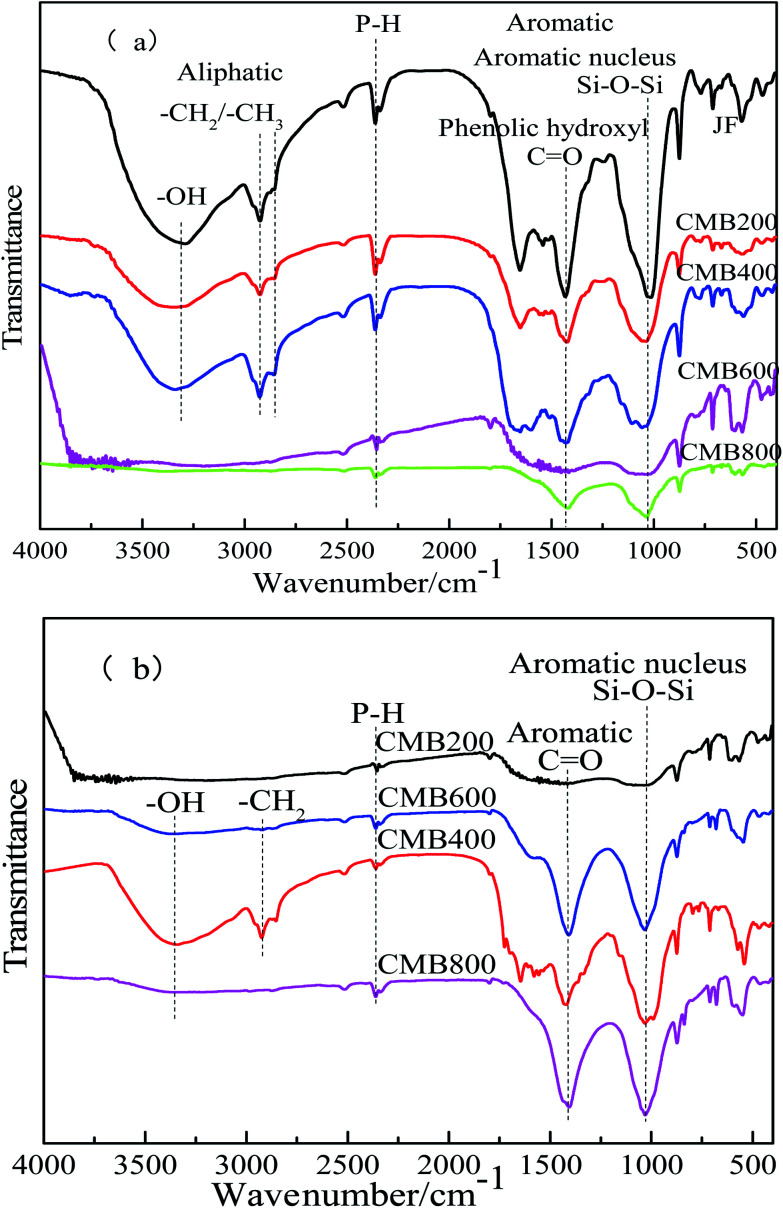
FTIR spectra of CMB before (a) and after (b) Pb^2+^ reaction.

The FTIR spectra can be used to investigate the functional groups contained in a material. Kumar *et al.* confirmed the presence of the hydroxyl (–OH), carboxylic (–COOH), and carbonyl (–C=O–) functional groups in biochar using FTIR spectra.^44^ The FTIR spectra of the CMB samples following pyrolysis at 200, 400, 600, and 800 °C are shown in Fig. 5. The FTIR spectra of the CMB samples before and after being treated with Pb^2+^ are shown in Fig. 5a and b, respectively. Before Pb^2+^ adsorption, the intensity of absorption peaks diminishes with increasing carbonization temperature, however, the opposite is true after Pb^2+^ adsorption.

The peaks between 3292 and 3380 cm^−1^ represent hydroxyl (O–H) bond stretching vibrations. The peaks between 2855 and 2926 cm^−1^ represent C–H (methylene –CH_2_) bond stretching vibrations (Fig. 5a).^45^ Vibrations between 1659 and 1663 cm^−1^ can be attributed to the C=N stretching, although this was not apparent after Pb^2+^ adsorption (Fig. 5b). However, samples CMB600 and CMB800 did not show hydroxyl and alkanes peaks in these regions; loss of O and H functionality with the increase of pyrolysis temperature may result from decarbonylation, decarboxylation and dehydration reactions.^46^ O and H contents were declined sharply compared to the loss of carbon content, leading to the growth of carbon content in biochar with increasing temperature^47^ (Fig. 5a). Higher carbonization temperatures are reportedly unfavorable for the existence of hydroxyl and alkane groups.^28^

The region between 1056 and 1014 cm^−1^ represents the Si–O–Si stretching vibration. The intensity of the absorption peaks was greatest at higher carbonization temperature before treatment with Pb^2+^ (Fig. 5a). The Si–O–Si stretching vibration peaks are not apparent in Fig. 5b (after adsorption). The peaks between 1422 and 1452 cm^−1^ represent stretching vibrations of conjugated C–C bonds of aromatic rings, with all samples (CMB200–CMB800) showing this absorption peak.

## 4 Conclusions

The properties of the resultant CMB samples were significantly affected by pyrolytic process temperature. The Pb^2+^ ions adsorption capacity was increased with increasing pyrolytic temperature. The adsorption data shows that the pseudo-second order simulated kinetic data are close to the actual adsorption data, whereby the goodness of fit was better than that obtained from pseudo-second-order kinetics, thus accordingly, the pseudo-second order model provided a better fit for the entire kinetic run. The chemical adsorption process dominated. The Langmuir model fitted the sorption isotherm better than the Freundlich model. The sorption capacities were enhanced with increasing pyrolytic temperature and adsorption temperature. This shows that for the adsorption of Pb^2+^, chemical adsorption dominated, and that adsorption was similar to that of monolayer adsorption. The CMB samples derived from the highest pyrolysis temperature had the greatest adsorption capacity in comparison to that of a high pyrolysis temperature at all solutions for all pH conditions, and that the best adsorption effect of CMB for Pb^2+^ is in pH of 4.5–7.5 solutions.

## Conflicts of interest

The authors declared that they have no conflicts of interest to this work.

## Supplementary Material

RA-010-C9RA08199B-s001
