# Supplementary material for: Effect of different pyrolysis temperatures on physico-chemical characteristics and lead(ii) removal of biochar derived from chicken manure
Source: RSC Adv. 2020 Jan 22;10(7):3667–74. doi: 10.1039/c9ra08199b (PMC9048431; doi:10.1039/c9ra08199b)
Supplement: RA-010-C9RA08199B-s001 [file RA-010-C9RA08199B-s001.pdf]

Electronic Supplementary Material (ESI) for RSC Advances.
This journal is © The Royal Society of Chemistry 2020

Supplementary materials.

Table S1 EDS elemental composition analysis tables of CMBs before and after Pb^{2+} reaction.

	Sample	C	N	O	Ca	K	Pb
before reaction	CMB200(a)	9.03	2.41	20.36	65.75	1.44	/
	CMB400(b)	14.57	3.87	15.14	61.04	5.38	/
	CMB600(c)	14.78	17.49	10.1	57.63	/	/
	CMB800(d)	43.42	3.39	13.76	31.71	7.68	/
after reaction	CMB200(e)	5.63	12.09	23.41	26.7	/	4.88
	CMB400(f)	19.47	4.69	15.43	48.38	/	10.55
	CMB600(g)	41.98	16.85	10.79	23.82	/	16.56
	CMB800(h)	18.32	4.38	8.78	62.33	5.84	20.30
